# Novel Cell and Tissue Acquisition System (CTAS): Microdissection of Live and Frozen Brain Tissues

**DOI:** 10.1371/journal.pone.0041564

**Published:** 2012-07-24

**Authors:** Lili C. Kudo, Nancy Vi, Zhongcai Ma, Tony Fields, Nuraly K. Avliyakulov, Michael J. Haykinson, Anatol Bragin, Stanislav L. Karsten

**Affiliations:** 1 NeuroInDx, Inc., Signal Hill, California, United States of America; 2 Division of Neuroscience, Department of Neurology, Los Angeles Biomedical Research Institute at Harbor-University of California Los Angeles (UCLA) Medical Center, Torrance, California, United States of America; 3 Department of Neurology, David Geffen School of Medicine at the University of California Los Angeles, Los Angeles, California, United States of America; 4 Department of Biological Chemistry, David Geffen School of Medicine at the University of California Los Angeles, Los Angeles, California, United States of America; Centro de Investigación en Medicina Aplicada (CIMA), Spain

## Abstract

We developed a novel, highly accurate, capillary based vacuum-assisted microdissection device CTAS - Cell and Tissue Acquisition System, for efficient isolation of enriched cell populations from live and freshly frozen tissues, which can be successfully used in a variety of molecular studies, including genomics and proteomics. Specific diameter of the disposable capillary unit (DCU) and precisely regulated short vacuum impulse ensure collection of the desired tissue regions and even individual cells. We demonstrated that CTAS is capable of dissecting specific regions of live and frozen mouse and rat brain tissues at the cellular resolution with high accuracy. CTAS based microdissection avoids potentially harmful physical treatment of tissues such as chemical treatment, laser irradiation, excessive heat or mechanical cell damage, thus preserving primary functions and activities of the dissected cells and tissues. High quality DNA, RNA, and protein can be isolated from CTAS-dissected samples, which are suitable for sequencing, microarray, 2D gel-based proteomic analyses, and Western blotting. We also demonstrated that CTAS can be used to isolate cells from native living tissues for subsequent recultivation of primary cultures without affecting cellular viability, making it a simple and cost-effective alternative for laser-assisted microdissection.

## Introduction

Isolation of specific anatomical regions and cells from complex heterogeneous tissues is a prerequisite step for understanding cell specific regulatory mechanisms in both health and disease. The mixtures of different cell types result in “averaging out” of results, masking disease specific changes often pronounced only in specific subanatomical regions or cell types. This is a particularly important issue in neuroscience where brain tissues demonstrate incredible complexity and a disease often affects only particular brain regions or cell types, posing a remarkable challenge for understanding basic brain functions or in the drug discovery process [1], [2]. Therefore, procurement of pure cell populations from a heterogeneous tissue sample is a prerequisite for sound molecular studies. Tissue processing for molecular studies usually involves some or all of the following steps: tissue collection, gross dissection/identification, fixation and processing, staining, and microdissection for downstream analyses of isolated cellular populations that involves a range of methods, including genomics and proteomics studies. Cell and region specific studies require highly reliable technologies allowing the separation of specific cell types from normally heterogeneous tissues such as the brain. Technically the most important parameters of tissue microdissection are precision, prevention of contamination, efficiency, and cellular resolution, which determine the quality of the collected samples for downstream analyses. There are several techniques ranging from manual microdissection to laser-assisted technologies, including laser ablation [3], [4], [5], laser pressure catapulting [6], [7] and laser capture microdissection [8], [9], [10]. Microdissections have been performed using techniques, such as micropunching [11], [12], but no standardized instrument has been developed aside from laser-assisted microdissection devices.

Manual tissue dissection is usually performed on routinely stained slides using 5- to 100-µm-thick sections placed on non-coated glass slides. It is time-consuming, operator dependent and has a high risk of contamination. Laser-assisted microdissection techniques can help to cope with high tissue complexity, reduce risks of contamination and increase reproducibility of cell procurement. Laser capture microdissecting (LCM) is currently the most popular technology that has been applied widely in the past two decades [13], [14], [15], [16]. Laser-assisted techniques are typically performed on fixed tissues and therefore can’t effectively use native tissues for subsequent cell culturing and further *in vitro* experimentation. Such capability would be especially useful for the stem cell field where spatially separated and often functionally specialized populations of stem cells and progenitors are the research target [17], [18], [19], [20], [21]. Furthermore, laser-assisted microdissection technologies often involve heat and irradiation process (e.g. laser ablation or UV laser cutting), which may affect the quality of macromolecules, such as RNA and proteins, increasing the risk of experimental variability and artifact generation in downstream analyses [22]. Recent studies reported that tissue pretreatment prior to LCM may decrease the RNA quality by more than 30% [23], [24]. Another limitation of laser-assisted microdissection instruments is the prerequisite use of thin tissue sections of 10 to 20 µm that results in the use of multiple tissue sections for the collection of a large sample volume. In addition, laser-assisted microdissection instruments are usually very expensive, limiting accessibility to these technologies for many research groups. With a growing demand for the development of novel cell- and tissue-specific diagnostic and therapeutic approaches, there is a tremendous need for a simple-to-use and cost-effective microdissection instrument capable of achieving resolutions comparable to laser-assisted instrumentation and applicable to a wider range of parameters, including both fixed and native tissues, as well as thicker tissue sections.

Here we describe a new low-cost capillary-based vacuum-assisted cell and tissue acquisition system (CTAS) capable of collecting subanatomical regions, cell clusters and specific cells from native (live), fresh-frozen, and sucrose treated brain tissues. CTAS is attached to an inverted microscope and microdissects tissue sections under direct microscopic visualization. It has been validated in both fresh frozen and native central nervous system (spinal cord and brain) tissues and the quality of macromolecules isolated from CTAS samples has been evaluated. Collected samples are suitable for primary cell cultures and extraction of high quality RNA, DNA and proteins for downstream applications, such as cell type specific genomic or proteomic analysis.

## Methods

### Cell and Tissue Acquisition System (CTAS)

**Figure 1:**
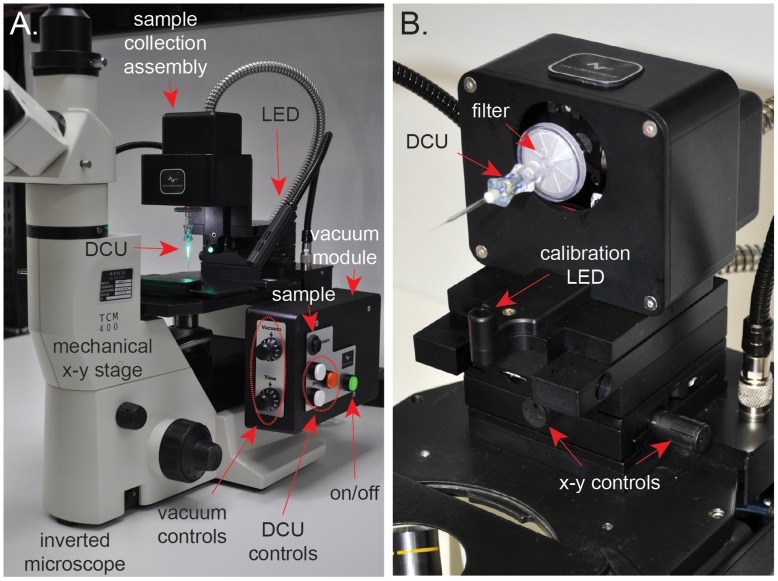
Capillary-based vacuum-assisted cell and tissue acquisition system (CTAS) v.4.1. **A.** Representative image of CTAS v.4.1. The system is attached to an inverted microscope (TCM400, Labomed) and consists of the following key components: sample collection assembly with collection unit, LED lights, side chassis vacuum module and DCU controls. The latter incorporates electronic controls and a vacuum pump. The two dials at the front of the side chassis control the vacuum strength from 2″Hg (1) to maximum of 22″Hg (10) and the vacuum duration from 100 ms (1) to maximum of 1 second (10). Depending on the tissue type and section thickness, various vacuum strength and duration may be used. Green button turns the power on/off. Three DCU control buttons include two white buttons, which bring the DCU up or down during the calibration procedure and an orange button that sets the Home position of the DCU and brings it to its Standby position. Black “Sample” button initiates sample collection by bringing the DCU down to the Home position and activating the vacuum at the selected strength and duration; **B.** CTAS sample collection assembly in its lifted position for DCU attachment/removal. DCU attached to a collection unit with connectors for multiple cables and a vacuum tube. Calibration LED source for illuminating the tip of the capillary is shown. In this position, the green horizontal and red vertical calibration LED lights are automatically turned off. The lights are automatically turned on when the CTAS head is in its upright position. The x–y position of the DCU is controlled by the knobs on the linear stages (x–y controls).

CTAS v.4.1 is attached to an inverted TCM400 (Labomed) microscope and consists of the following major components: sample collection assembly, vacuum module and controls, two gooseneck LED lights and single axis stages (Fig. 1). A linear actuator is used for the vertical movement of the disposable capillary unit (DCU), providing precise positioning during calibration and sample acquisition (Fig. 1). DCU is connected to the vacuum line through the disposable filter unit (Millipore; Fig. 1). LED lights (Littlite) are used to illuminate the slide with a tissue sample (Fig. 1). Vacuum module incorporates electronic controls and a vacuum pump with pneumatic tubing (Fig. 1). Vacuum pump vibration mount ensures that linear actuator stability is not affected during pump operation. Two distinct vacuum pumps are used for the basic microdissection CTAS model v.4.1 (KNF Neuberger, MPU2568-NMP830-10.10) and CTAS-live tailored for isolation of live brain cells (KNF Neuberger, UN86KNDCB). Pump generating a stronger vacuum is required for the microdissection of generally thicker native tissues. Our current CTAS electrical circuit board is programmed to provide an appropriate range of the variable parameters for vacuum strength, duration and LED lights intensity. It incorporates the controls for the vertical movement of the linear actuator and positioning the tip of the DCU relative to the sample - calibration procedure (Fig. 1). The single-axis stages are used for positioning the DCU in the center of the microscope’s x-y stage (Fig. 1A–C). The outer casing of the CTAS components were designed in Solidworks and machined of aluminum alloy. The inverted microscope is equipped with a trinocular port for mounting a camera and/or video system with an appropriate adapter for process documentation or visualization via a computer monitor.

### Disposable capillary unit (DCU)

**Figure 2:**
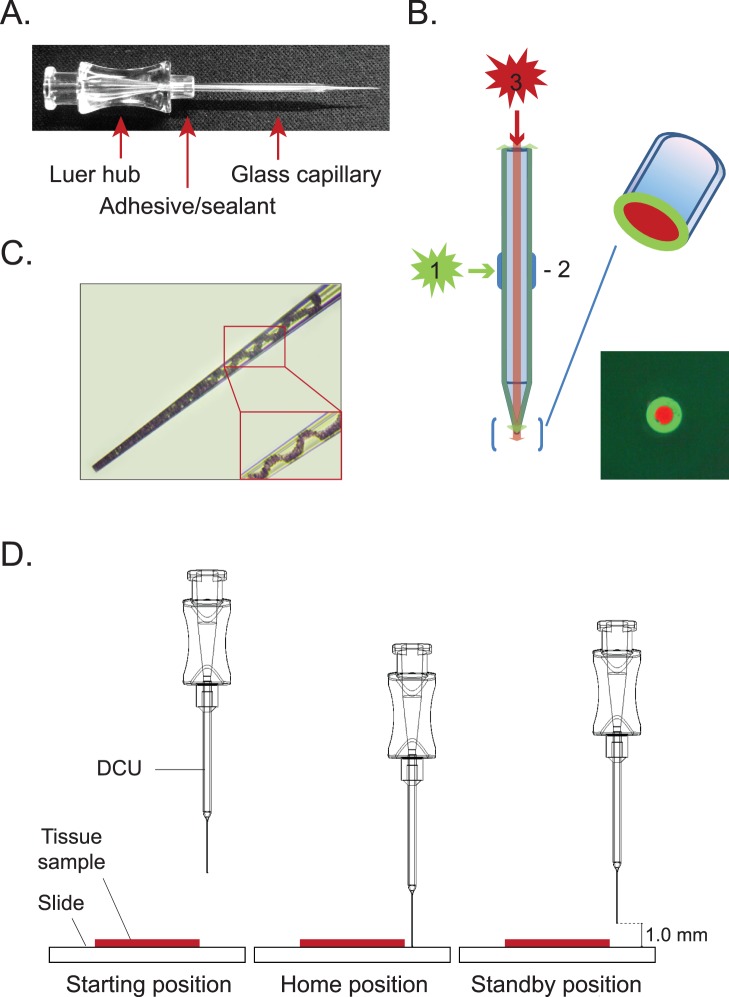
Disposable capillary unit (DCU) and its positions during calibration and sample collection. **A.** DCU consists of a Luer hub, connected to a glass capillary with an adhesive. **B.** The tip of the capillary is illuminated by the reflected light from the orthogonal light source (1) directed onto a treated or coated surface of the glass capillary barrel (2). To enhance this effect, additional light source (3) maybe used above the upper capillary opening to produce better visualization of the low capillary tip end. Lower images shows the actual microscope visualization of the capillary tip (OD = 20 µm). **C.** Calibration procedure involves three DCU positions: starting position prior to calibration, home position when the DCU tip is in contact with tissue section and standby position when the DCU tip is lifted above the tissue sample and ready to perform multiple acquisitions; **D, E.** Tissue sample in the barrel of the capillary. OD = 50 µm.

DCU consists of a pulled glass capillary with a preset diameter coupled to a Luer hub (Qosina) which is connected with the main vacuum line during a dissection (Fig. 2A). To prevent possible cross contamination during sample collection, a disposable filter unit (Millipore) is incorporated above the DCU (Fig. 1D). The DCU has an easy-to handle needle hub, which makes a sealed connection with the filter unit (Fig. 1B). Capillaries of various diameters are made from borosilicate glass using a micropipette puller manufactured by Sutter Instruments (model P-2000). Capillaries with 1.5 µm thick wall and 0.84 mm inner diameter (ID) are used to produce tips ranging from 1 µm to 200 µm ID. Because the manufacturing error of the tip ID is within ±5%, for quality control, the tips of the capillaries are examined under the microscope and polished, if necessary, using a microforge-grinding center (MDI, NJ). Capillary tip illumination for calibration purposes is achieved by the projection of a colored light from an orthogonal light source along the wall of the capillary (Fig. 2B). If necessary, an additional vertical light source can be used to enhance the visibility of the capillary tip during the calibration process (Fig. 2B). After sample collection (Fig. 2C), the DCU containing the dissected material and buffer is removed and placed into a standard 1.5 ml test tube into which the contents are released using a standard syringe.

### Animals and tissues

For this study, adult Wistar rats, adult FVB/N mice (6 to 9-months old) and embryonic (E18) C57 mouse brain tissues (BrainBits, LLC, IL) were used. The animal protocols were in accordance with the NIH Guide for the Care and Use of Laboratory Animals and were approved by the Los Angeles Biomedical Research Institute Animal Studies Committee (mice) and UCLA Animal Research Committee (rats). Sacrificed animals were perfused with PBS and CNS regions of interest were dissected. Tissues were either fresh frozen or sucrose treated and then frozen until further use. For sucrose treatment, tissue samples were sunk in 15% sucrose solution in PBS and kept overnight. Fresh frozen or frozen sucrose treated brains were sectioned into 10 µm to 50 µm sections, placed onto glass slides and either kept at −80°C or immediately used for CTAS microdissection. When necessary, sections were briefly (10–20 seconds) stained with 0.01% solution of Toluidine Blue O (Carolina Biological Supply Company) or with 0.2% Cresyl Violet (Sigma) and rinsed with ice cold PBS [25].

Live adult and embryonic mouse brain tissues (BrainBits, LLC, IL) were used for dissection of ventricular zones and subgranular layers to establish cultures of neural progenitors. Immediately after dissection adult brains were coronally sectioned using Leica VT1200 vibratome generating 300 to 500 µm thick sections in ice cold artificial cerebrospinal fluid (ACSF) containing 124 mM NaCl, 26 mM NaHCO_3_, 3 mM KCl, 1.25 mM NaHPO_3_, 2.0 mM MgCl_2_, 10 mM glucose, and saturated with 95% O_2_, 5% CO_2_, pH 7.4. The sections covered with ice cold HBSS (Hank’s Balanced Salt Solution) were immediately used for dissection. Embryonic live mouse brains were received from BrainBits, LLC preserved in Hibernate medium and used for isolation of ventricular zones.

### CTAS Acquisition Area Calculations

To estimate the tissue area acquired by CTAS during dissection, series of individual acquisitions (“picks”) were performed using mouse fresh frozen brain sections (neocortex) of various thickness. At least twenty individual acquisitions were made for each tissue thickness, DCU ID, vacuum strength and vacuum duration time. The size of acquired areas was measured using ImageJ [26] and the average acquisition area and standard deviation were calculated in Excel (Table 1). Based on the average acquisition area values, sample volumes were calculated and the numbers of “picks” were estimated for the collection of 0.5 mm^3^ of sample tissue.

**Table 1:** Estimation of mean acquisition areas for various DCU IDs and tissue thicknesses using neocortical sucrose treated mouse brain sections.

DCU tip ID (µm)	Tissue thickness (µm)	Vacuum duration (s)	Vacuum strength (Hg”)	Mean sample area (mm^2^)	STDEV (mm^2^)
20	20	0.5	8.8	0.001	0.000
20	20	1.0	17.6	0.004	0.001
40	20	0.1	17.6	0.013	0.002
40	20	0.5	22	0.013	0.005
40	20	1.0	4.4	0.010	0.004
50	20	0.5	22	0.018	0.005
50	50	1.0	2.2	0.012	0.005
50	50	0.5	17.6	0.010	0.003
90	20	1.0	4.4	0.035	0.005
90	20	0.1	11	0.057	0.004
90	50	0.1	8.8	0.062	0.005
90	50	0.5	8.8	0.093	0.013

### RNA isolation and analysis

Total RNA was isolated from dissected CTAS collected tissue material using either acid phenol extraction (Trizol LS; GIBCO/BRL) or Qiagen RNeasy Micro Kit, according to manufacturers’ instructions. The concentration and 260/280 ratios were measured using Nanodrop Spectrophotometer. The integrity of RNA was evaluated using Agilent Bioanalyser 2100. For evaluation of RNA integrity at least 3 independent tissue samples (n≥3) were collected for each time point and tissue type.

### 2D gel electrophoresis analysis

Protein extracts were prepared from CTAS dissected tissue samples isolated from the neocortex, hippocampus, CA3 and dentate gyrus (DG) areas of the mouse brain. Collected tissues samples were briefly centrifuged at 13,500 rpm at 4°C to separate solid tissue fragments and a liquid portion. Solid tissue portion was dissolved in labeling buffer (7 M urea, 2 M thiourea, 4% CHAPS and 25 mM Tris-HCl, pH 8.5) and then sonicated briefly at 4°C. Resultant protein extracts and liquid portions were precipitated separately using 2D Clean-up kit (GE Healthcare), both protein pellets were resuspended in the labeling buffer, centrifuged at 13,500 rpm at 4°C for 10 min, and both supernatants were combined. Protein concentration was determined using 2D Quant kit (GE Healthcare). Protein samples from DG and CA3 areas were labeled correspondingly with the *N*-hydroxysuccinimidyl ester derivatives of Cy3 and Cy5 dyes according to standard labeling protocol (GE Healthcare). After labeling both protein samples were mixed together (12.5 µg each), rehydration solution was added (7 M urea, 2 M thiourea, 4% CHAPS, 1% DTT, 0.5% non-linear pH 3–11 IPG buffer, 5% glycerol, 10% isopropanol), samples were applied to 13 cm pH 3–11 nonlinear IPG strip (GE Healthcare), and isoelectric focusing was performed for the total of 17,000 V/hrs. After isoelectric focusing proteins were separated by SDS gel electrophoresis using 8–16% gradient gel (Bio-Rad). Gel images were scanned on the Typhoon Trio Variable Mode Imager (GE Healthcare) at 50 micron resolution using 532 nm laser/580BP30 nm filter for Cy3 and 633 nm laser/670BP30 nm filter for Cy5. Gel images were analyzed using the Decyder 2D Differential Analysis software v. 6.5 (GE Healthcare). Gels were fixed and stained by Sypro Ruby; protein spots were manually picked, and digested with trypsin. Mass spectrometry analysis was performed as previously described [27].

### Neural progenitor cultures and immunocytochemistry

Floating neural progenitor cultures (neurospheres, NS) were established as previously described [28] with minor modifications. The tissue was transferred into dissociation solution containing 0.05% trypsin and 1 mM EDTA, followed by gentle trituration using fire polished pipette. The tissue was incubated for 15 minutes, triturated, incubated for another 10 minutes and washed twice with HBSS by centrifugation (1,500 rpm; 10 min). The neural progenitor cells were plated in Neurobasal media with supplements, epidermal growth factor (EGF) and fibroblast growth factor (bFGF) (Life Technologies) as previously described [29]. Spherical cell clusters, neurospheres, were formed from each mouse, and when large spheres were noted (on average 10 days), the cells were briefly trypsinized and plated at a concentration of 2,500 cells per ml. For immunocytochemistry neurospheres were cultured on poly-L-lysine-coated coverslips. Immunocytochemistry was performed according to standard protocols with antibodies directed against Nestin (Rat-401, DSHB, IA) to visualize uncommitted progenitors [30], [31], [32] and glial fibrillary acidic protein (GFAP; Dako, CA) to identify astrocytes [33].

## Results

### CTAS Operation and Calibration

CTAS’s critical blocks are outlined in Figure 1. CTAS is attached to an inverted microscope and includes a DCU position module with a sample collection assembly at the top that moves the capillary tip up or down towards the stage and enables the calibration of DCU for cell and tissue acquisition; vacuum module that controls and provides adjustable vacuum strength and duration to dissect and capture the cells and tissue regions of interest; mechanical x-y stage for positioning of the sample in alignment with the capillary unit tip; and adjustable LED illuminators (Fig 1A). The overall working concept for CTAS is to dissect cells, cell clusters and tissue regions from tissue sections under direct microscopic visualization by applying negative pressure that is controlled by a vacuum system (Fig. 1). The tissue/cells of interest are located directly under the precisely calibrated DCU tip using the X–Y mechanical stage. Vacuum impulse produces the intake of the desired tissue section or individual cells. The cells/tissue sample is “vacuumed in” with some tissue stabilizing fluid (e.g. sterile buffer or culture medium) in to the “barrel” of the DCU (Fig. 1 and 2). After the desired amount of material is acquired into the DCU (Fig. 2C) it is transferred into a 1.5 ml test tube for DNA, RNA or protein isolation steps or a dish with culture medium for primary cell culturing. The 1.5 ml tube may contain the desired buffer for subsequent application, or the samples may be frozen for later use if appropriate.

Precise DCU tip positioning is calibrated with the position controller (Fig. 1), which makes the instrument ready for repeated tissue acquisition. Calibration is performed prior to every dissection experiment and normally takes several minutes. During calibration, the area of interest is placed near the center of the focusing light that denotes the position of the capillary – *starting position* (Fig. 2D). Using the DOWN positioning button the DCU is lowered until the tip comes in contact with the surface of the tissue section. A single quick press of either of the positioning buttons will move the DCU by 1.5 µm. When either of the white positioning buttons is held down for more than 5 seconds, the maximum speed will reach 3.5 µm/s. Pressing the HOME (orange) button establishes the *home position* (Fig. 2D), which will lift the DCU 1 mm above the tissue section to its *standby position*. DCU will be returned to the *standby position* after each acquisition to ensure unobstructed horizontal movement of the sample when moving to the next region of interest for acquisition (Fig. 2D). After calibration is completed, repeated collection of the desired tissue regions may be performed. Pressing the SAMPLE (black) button on the control panel (Fig. 1B) triggers the collection of the tissue sample by a vacuum impulse. A dissected cells/tissue sample is collected into the DCU’s capillary barrel (Fig. 2C). After the sample is collected and the DCU is returned to the *standby position*, the next area of interest can be acquired by repeating this procedure. The whole process is rapid, with each individual acquisition taking less than three seconds. Furthermore, with the trinocular model (Fig. 1), a digital microscope camera can be connected to a computer to operate CTAS while viewing the tissue image on the computer screen.

### Microdissection of Mouse Brain Subanatomical Regions

**Figure 3:**
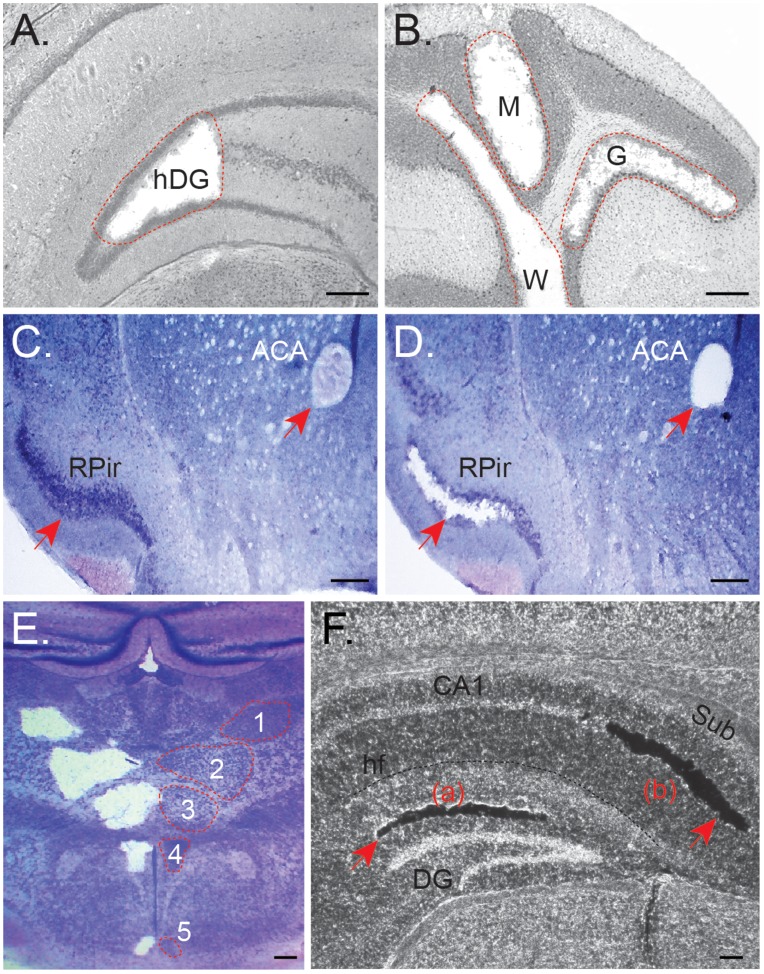
Dissection of subanatomical regions (marked with red dotted line or arrows) from fresh frozen mouse coronal brain sections (20 µm thickness) using CTAS v.4.1. A. Collection of the left hilus of the dentate gyrus (hDG). **B.** Dissection of granular cells (G), molecular layer (M), and white matter (W) from mouse cerebellum. Intact (**C**) and dissected (**D**) anterior commissure, anterior (ACA) and right piriform cortex (Rpir). **E.** Dissected (left) and intact (right) thalamic and hypothalamic areas including posterior thalamic nucleus (1), part of ventral posteromedial thalamic nucleus (2), ventromedial thalamic nucleus (3), dorsomedial hypothalamic nucleus (4) and arcuate hypothalamic nucleus (5). Homotopical intact areas are outlined with dashed red lines. Tissues were stained with Toluidine Blue. Scale bar  = 250 µm. DCU ID = 50 µm; vacuum pulse duration: 100 ms; **F.** Representative microdissection of middle molecular layer of the dentate gyrus (a) and cellular layer of the subiculum (b) from fresh frozen nonstained mouse brain sections (20 µm thickness). Abbreviations: CA1–CA1 area of hippocampus; Sub – subiculum; DG – dentate gyrus; hf – hippocampal fissure. Scale bar  = 100 µm.

**Figure 4:**
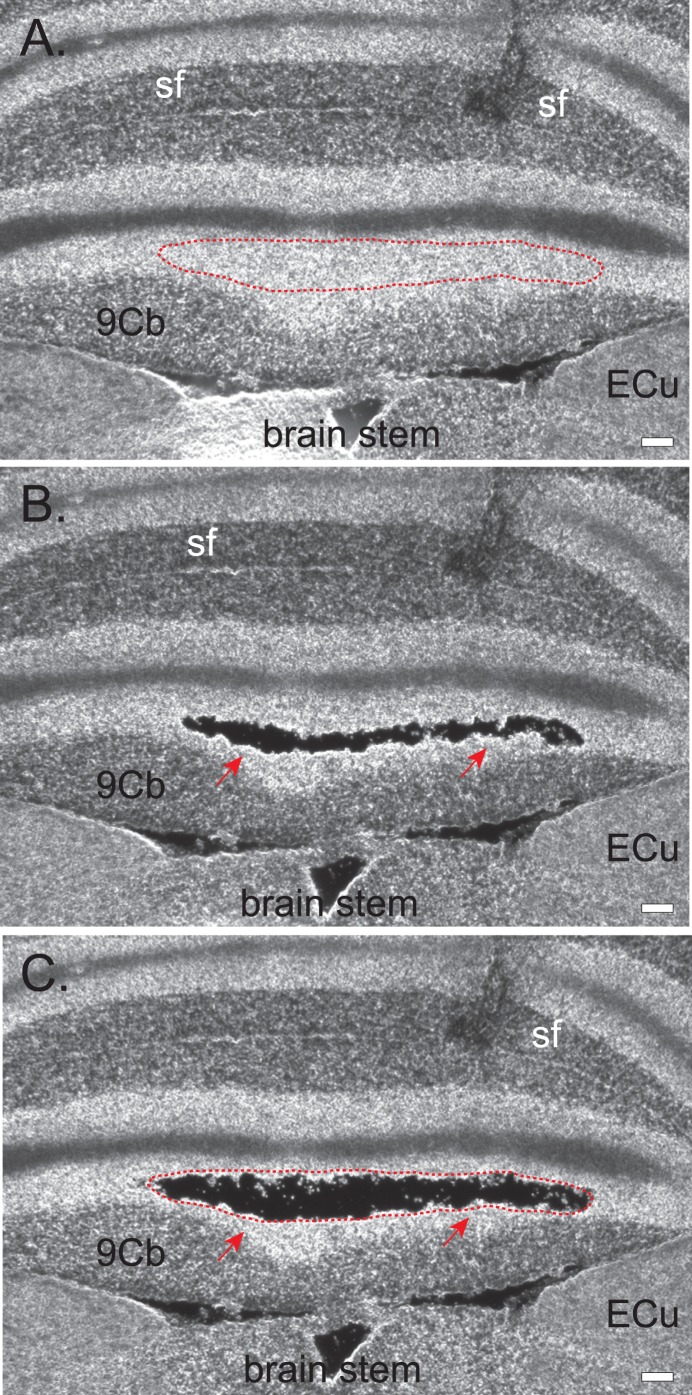
Sequential microdissection of granular cells from the 9^th^ lobe of cerebellum using fresh frozen and unstained moue brain sections (thickness  = 20 µm). A. Red dotted line outlines the area of interest. **B.** Dissection of granular cells from the middle part of the granular layer. **C.** Additional dissection from the same area. Abbreviations: Sf – the secondary fissure of the cerebellum; 9Cb –9^th^ area of cerebellum, Ecu – external cuneate nucleus [50].

To demonstrate that CTAS is capable of collecting desired tissue regions and cell clusters we performed a series of microdissections using mouse and rat brain tissues of various preparation types and thickness. Brain tissue was selected as the most heterogeneous and anatomically complex tissue type in the mammalian organism. Using CTAS we performed microdissection of fresh frozen and sucrose treated mouse and rat brain tissues and demonstrated its feasibility to precisely dissect desired subanatomical regions and cell clusters. Various tissue section thicknesses were tested and some of the suggested parameters are summarized in Table 1. The optimal range of tissue thickness was found to be between 10 µm and 300 µm depending on the preparation type. As shown in Fig. 3 and 4, the different subanatomical parts of the mouse brain were precisely dissected, including the hilus of the dentate gyrus (Fig. 3A), molecular layer, granular layers and white matter of cerebellum (Fig. 3B), anterior commissure, anterior and right piriform cortex (Fig. 3C–D), thalamic and hypothalamic areas including posterior thalamic nucleus, part of ventral posteromedial thalamic nucleus, ventromedial thalamic nucleus, dorsomedial hypothalamic nucleus and arcuate hypothalamic nucleus (Fig. 3E). Dissections were also performed on unstained sucrose treated (Fig. 3F) and fresh frozen tissues (Fig. 4). We also dissected different pyramidal layers of posterior CA3, posterior and ventral pyramidal layers of the hippocampus, as well as a mixture of granular and inner molecular layers of the dentate gyrus and lateral entorhinal cortex, individual layers of the motor cortex and piriform cortex (not shown). Using CTAS, different brain subanatomical regions could be collected from both stained and unstained fresh frozen and sucrose treated tissues (Fig. 3 and 4).

### Microdissection of Individual Interneurons and Purkinje Cells

**Figure 5:**
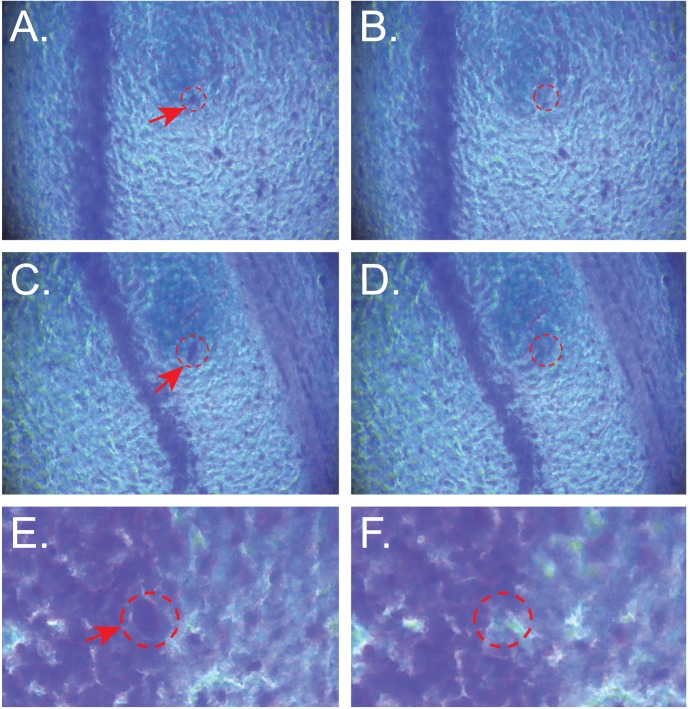
Representative collection of individual CA1 interneurons in stratum orience (A –**D) and Purkinje cells (E**–**F) from sucrose treated mouse coronal brain sections.** Images before (**A**, **C**, **E**) and after (**B**, **D**, **F**) collection are shown. Red arrows and dashed circles show collected cells. Tissue thickness  = 20 µm. DCU ID = 20 µm. Magnification: 400X.

To test the capabilities of CTAS for isolation of individual cells, we used frozen sucrose treated adult mouse coronal brain sections with 20 µm thickness. Interneurons from stratum radiatum and stratum orience and individual Purkinje cells were successfully isolated using ID tip 20 µm, vacuum strength from 11″ to 20″ Hg and vacuum duration from 0.3 to 0.5 seconds (Fig. 5).

### CTAS Operation Parameters, Acquisition Area and Dissection Time Estimates

The force of the tissue fragment intake depends on the cross sectional area of the glass capillary, tissue section thickness, properties of the extracellular matrix, and the power and duration of the vacuum applied. Vacuum duration and strength are adjustable for various tissue types, sample preparation methods, and tissue thickness. These parameters may be defined by the size of the cells or tissue sections being collected. Optimal vacuum levels and pulse duration may be determined using a test sample prior to actual sample collection. It is essential to keep the tissue moist throughout the microdissection procedure. DCUs with 20 µm to 50 µm ID work best for extraction of cell clusters or subanatomical areas such as dendritic layers in the hippocampus and cortical layers (e.g. dentate gyrus, medial entorhinal cortex; see Fig. 3 and 4). Capillaries with 10 µm to 20 µm ID are best for extraction of individual cells (e.g. Purkinje cells; see Fig. 5). Capillaries with ID of 50 µm to 100 µm can be used for collection of larger subanatomical regions (different nuclei in the thalamus, brain stem or olfactory bulb, ventricular zones) using sucrose treated, fresh frozen (Fig. 3 and 4) and native tissues.

**Figure 6:**
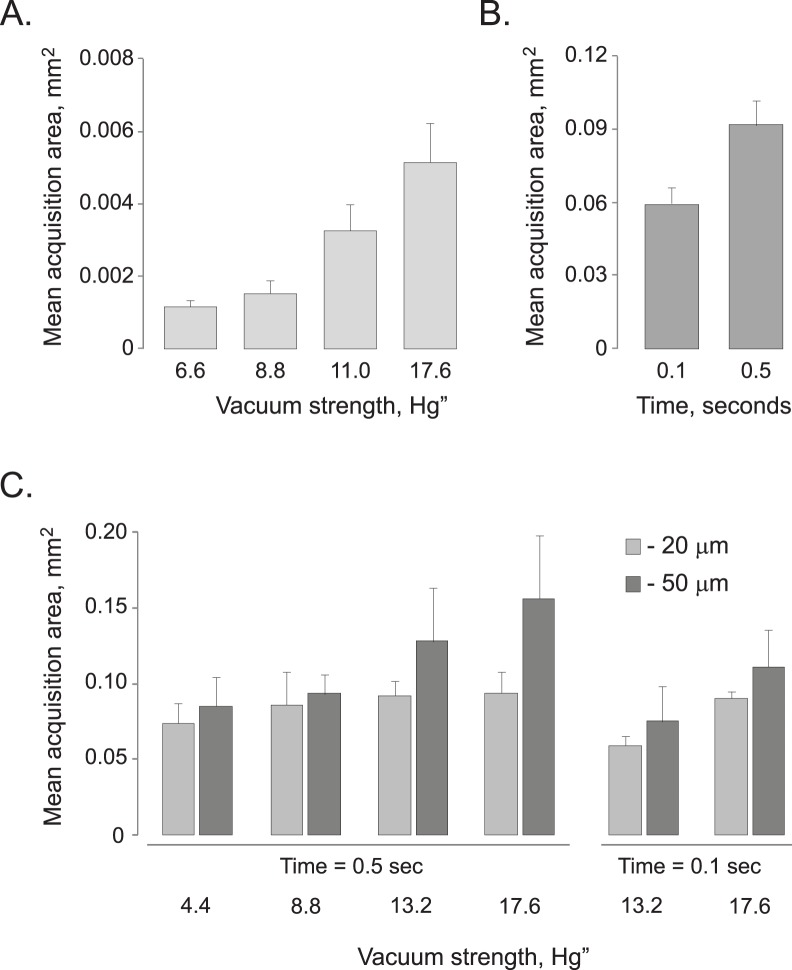
Representative charts demonstrate the dependence of average acquisition area on the vacuum strength, vacuum duration and tissue thickness. Adult mouse coronal neocortical tissue sections were used. As expected, increased vacuum strength and duration, as well as thicker tissues, negatively affect the accuracy of the dissection producing larger acquisition areas with higher variability. At least 5 individual acquisitions (n≥5) were made to estimate mean acquisition area for each parameter tested. Error bars represent standard deviations; **A.** Mean values of acquisition area for 20 µm tissue and 20 µm DCU ID. Notice the proportional increase in the average area with the increase in the vacuum strength; **B.** Longer vacuum duration produces larger acquisition area. Tissue thickness: 20 µm, DCU ID = 90 µm; **C.** For the same vacuum strength and duration, thicker tissues produce larger and more variable acquisition areas compared to thinner sections. Comparisons were made for 0.5 sec (left) and 0.1 sec (right) time duration. Tissue thickness 20 µm (light grey) and 50 µm (dark grey), DCU ID = 90 µm.

Although, it is difficult to derive “universal” microdissection parameters, as they will depend greatly on the type of tissue used, we attempted to approximate the average sample area for several different DCU IDs and tissue thicknesses. Serial acquisitions (n>20) of neocorical areas were performed on mouse coronal tissue sections of various thickness using a range of vacuum strength and duration. The resulting sample acquisition areas are summarized in Table 1. It is important to mention that the acquisition area size and associated variability gradually increased with increasing vacuum strength (Fig. 6A) or vacuum duration (Fig. 6B) for all DCU IDs and tissue thicknesses. Acquisition area size and variability were generally higher for thicker tissues when the same DCU ID was used (Fig. 6C). Both observations were not unexpected and once again emphasize the importance of proper parameter determination prior to the dissection experiment.

When parameters are optimized, an area of the acquired tissue sample slightly exceeds the area of the DCU tip (Table 1). Therefore, knowing the tissue thickness, average sample area per acquisition, and the amount of RNA or protein material required for a particular assay, it is straightforward to calculate the approximate number of individual acquisitions (“picks”) necessary to obtain a particular amount of sample material. Our data show that a collection of 0.5 mm^3^ of tissue sample from 50 µm thick brain tissue section using DCU with 90 µm ID would require 50 to 500 individual “picks” depending on the vacuum strength and duration. Assuming that each “pick” requires 3 seconds, the total dissection time should be under 30 minutes.

### Isolation of High Quality RNA and Protein from CTAS-dissected Tissues

**Figure 7:**
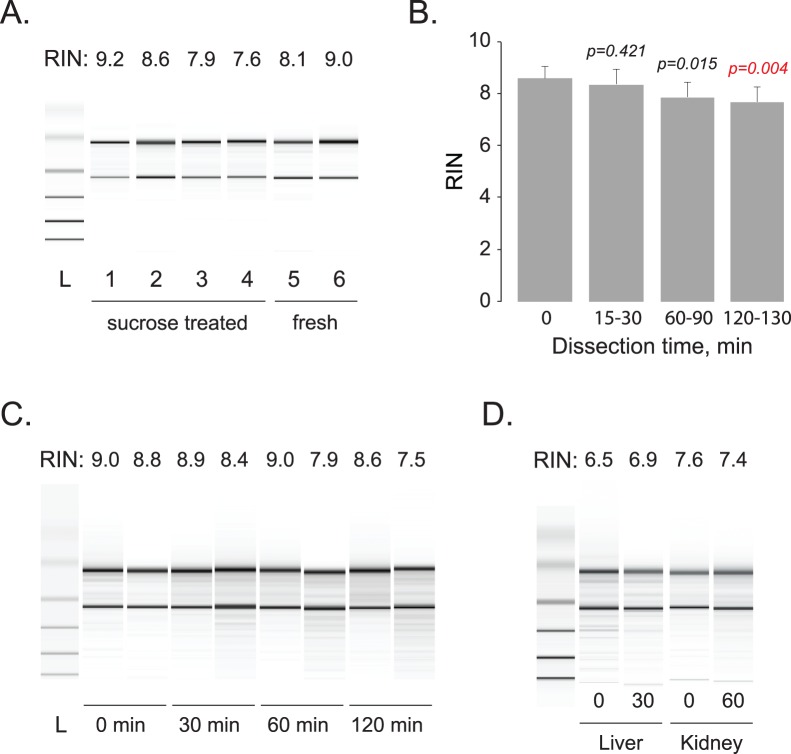
Total RNA isolated from CTAS microdissected samples shows good integrity. A. Representative quality of total RNA isolated from sucrose treated (1–4) and fresh frozen (5–6) mouse (1–2, 5–6) and rat (3–4) brain tissue samples. RNA integrity numbers (RIN) are shown for each sample. **B.** Mean RNA integrity numbers (RIN; n≥3) over dissection time show slow decline over time but remain within acceptable range for further analysis. Only 120–130 minutes time point demonstrate significant difference from RNA isolated immediately. **C.** Representative quality of total RNA isolated from brain tissues (mouse cortex) at different time points. **D.** Representative RNA isolated from liver and kidney tissues using CTAS based procedure demonstrates acceptable RNA quality after 30 minutes (liver) and 60 minutes (kidney) of microdissection.

**Figure 8:**
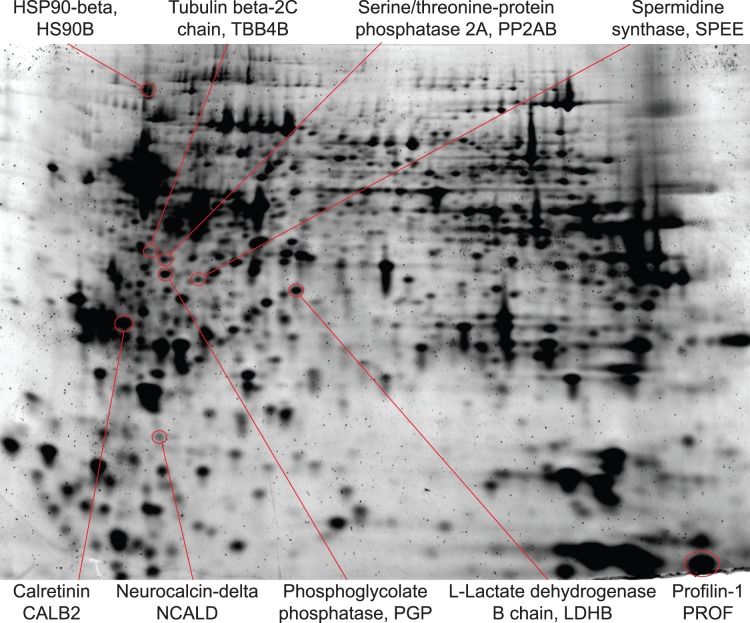
Proteins isolated from CTAS-dissected tissue samples preserve high integrity and may be used for downstream applications including 2D gel electrophoresis. Representative 2D gel electrophoresis analysis of fresh frozen CA2–CA3 and DG areas of hippocampus (stained by Sypro Ruby) is shown. Protein samples from CA2–CA3 and DG areas were labeled with Cy3 and Cy5 correspondingly, imaged on the Typhoon, and analyzed using DIA module of Decyder. Gel was fixed and stained with Sypro Ruby; protein spots were picked and identified using MS. The following differences in protein abundances between identified proteins isolated by CTAS from CA2–CA3 and DG areas were observed (CA2–CA3/DG abundances ratios are shown in parenthesis): HS90B (−1.52), TBB4B (−2.34), PP2AB (−1.55), PGP (used as control, no difference), SPEE (−1.59), LDHB (used as control, no difference), CALB2 (1.76), NCALD (1.77), PROF1 (1.65).

One of the main concerns for all microdissection techniques is the integrity of RNA molecules used for downstream comparative analyses such as real time PCR or DNA microarrays. To verify RNA quality, total RNA was extracted from CTAS-collected tissue samples acquired from sucrose treated and fresh-frozen mouse brain tissues, as well as fresh frozen mouse liver and kidney (Fig. 7). Independent of tissue preparation method, brain samples collected within 0.5 to 2.0 hours consistently demonstrated high RNA integrity with RIN above 7.5 when evaluated using Agilent Bioanalyzer 2100 (Fig. 7B–C). Kidney samples also showed consistently high quality RNA with high RIN values within 1 hour, and liver samples had expectedly slightly lower RIN values above 6.5 within 30 minutes (Fig. 7D). Total RNA yield from CTAS collected samples was in the expected range of 1.0 to 2.0 µg from 1 mg of wet brain tissue for either Trizol (Life Technologies) or RNeasy kit (Qiagen). To investigate the quality and integrity of protein samples dissected using CTAS, we extracted proteins from the fresh frozen dentate gyrus (DG) and CA2–CA3 tissue areas of adult mouse brain. Proteins extracted from the DG area were labeled with Cy3 and proteins from CA3 areas Cy5 dyes, correspondingly, mixed together and analyzed using 2D gel electrophoresis (Fig. 8). After electrophoresis and scanning on the Typhoon, fluorescently labeled protein images were analyzed using differential in-gel analysis (DIA) module of DeCyder software, which detected 1,613 spots corresponding to individual protein species resolved on the gel. According to DIA, the majority of proteins (97%) in both CA3 and DG samples have the same relative abundances in both tissue types. We also observed differential protein abundances for several protein spots. Some of the differentially expressed proteins were picked from the gel and identified using mass spectrometry (MS) (Fig. 8). Protein identification was performed using the Peptide Mass Fingerprinting (PMF) method and TOF/TOF sequencing of peptides. The PMF results showed that observed peptides span the whole length of the analyzed proteins, pointing to the maintenance of their integrity (1). Several examples are HS90B (23% coverage, 12 matched peptides), CALB2 (32%, 7 peptides), SPEE (26%, 7peptides) and PP2AB (36%, 8 peptides) (Dataset S1). Three identified proteins enriched in CA2–CA3 area corresponded to Calretinin (Calb2; 1.8 fold), Neurocalcin-delta (Ncald, 1.8 fold) and Profilin-1 (Pfn1; 1.65 fold). All three of the identified proteins were previously shown to have higher expression levels in the stratum oriens and pyramidal layer of CA2–CA3 areas compared to the dentate gyrus in line with our findings (Allen Mouse Brain Atlas; [34], [35]). MS analysis has shown that molecular weights and isoelectric points (pI) of all identified proteins accurately matched their position on the gel. We did not detect any evidence of protein degradation; hence we concluded that the quality of the samples isolated by CTAS from tissue sections is sufficient for proteomics studies.

### Neural Progenitor Primary Cultures from CTAS-dissected Tissues

**Figure 9:**
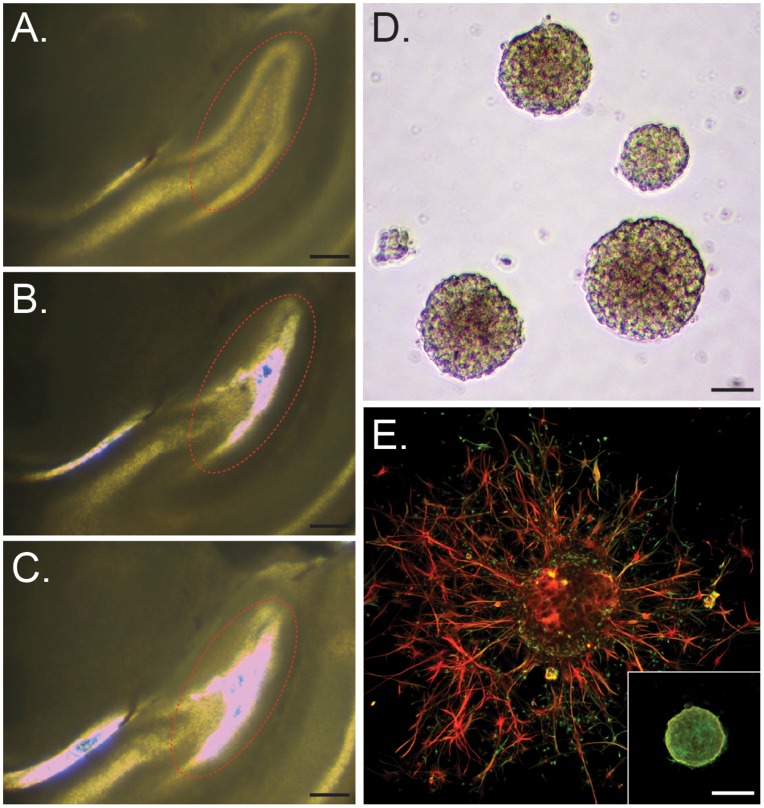
Collection of the neurogenic zones from native mouse brain tissues and neural progenitor cultures using CTAS-live. A–C. Representative images of subgranular layer of dentate gyrus collection from native adult rat brain tissue before (**A**), during (**B**) and after dissection (**C**). Tissue thickness  = 300 µm, Scale bar  = 250 µm; **D.** Phase contrast images of neurosphere colonies derived from E18 rat subventricular zone (SVZ). Partially differentiated (**E**) and undifferentiated (lower right inset) neurosphere (NS) colonies immunostained for nestin (green) and GFAP (red), the marker of neural progenitors and astrocytes, respectively. Scale bar  = 100 µm.

To demonstrate the utility of the proposed approach for efficient cultivation of primary neural progenitor cells (NPCs) derived from live brain tissues, we used CTAS-live featuring a stronger vacuum pump to collect subventricular zones (SVZ; Fig. S1) and subgranular layers (SGL; Fig. 9A–C) from live embryonic and adult rat brains and used them to establish neurosphere (NS) cultures. Live 300 µm to 500 µm brain tissue sections were used. Spherical cell clusters, neurospheres, were formed after each dissection. After culturing for 10 days in the presence of EGF and bFGF neurospheres were used for immunocytochemistry with antibodies directed against Nestin and GFAP. Intense staining for Nestin was observed in the floating and partially differentiated NS colonies (Fig. 9E). This demonstrated that developed *CTAS-live* is capable of acquiring live cells from native brain tissues. The straightforward protocol significantly reduces dissection time thus minimizing contamination and cell death.

## Discussion

Here, we developed an efficient, simple and cost-effective technology that permits tissue microdissection with similar capabilities to laser-assisted microdissection. Unlike laser-assisted methods working in the range of tissue thickness from 4 µm to 15 µm [36], CTAS works in a wider range of parameters permitting the dissection of tissue sections with various thicknesses ranging from 10 µm to 500 µm. Acquisition of specific subanatomical regions, cell clusters and individual cells was demonstrated in the mouse and rat brain tissues using both fresh frozen and sucrose treated tissues. CTAS’s capability to procure samples from thicker tissue sections permits rapid collection of large amounts of experimental material often required for the downstream protein studies. In addition, the ability of CTAS to microdissect thicker tissue sections permits the acquisition of live cells from native brain tissues that allows their subsequent culturing and further experimentation.

Acquisition of tissues and cells from precise anatomical location is a prerequisite experimental step towards our understanding of their function in both health and disease. This is especially important for preclinical translational research where disease often affects only specific cell types or tissue regions (e.g. dementia, cancer). Although several microdissection techniques have been developed in the recent past, certain limitations in this field still exist. Aside from the relatively high cost of laser-assisted microdissection instruments, other features prohibiting their wide spread application include the use of specific tissue preparation methods that may affect the quality of the macromolecules, inability to work with thicker tissue samples resulting in a very time consuming procedure when large amounts of sample material are required. In addition, its inability to microdissect live tissues limits the applicability of laser-assisted technologies for *in vitro* studies of primary cell cultures.

Harsch et al. developed an alternative microdissection approach that utilizes an ultrasonically oscillating needle [37]. The system applies a sharp steel needle to dissect areas of interest by longitudinal vibrations induced by a piezoelectric actuator, and the dissected tissues are collected by a micropipette. However, the accuracy is compromised due to the lateral needle vibration. Moreover, the procedure is complex in operation and does not allow dissection of live tissues. Other means of isolating live cells for primary cultures include flow sorting, a well established technology capable of separating a heterogeneous suspension of cells into purified fractions on the basis of fluorescence and light scattering properties [38]. Cell sorting is well suited for suspension of cells, such as hematopoietic cells or cell cultures, but in spite of rare successful attempts [39], is not an appropriate method for cell specific collection from adult tissue samples. Further, the invasive nature of tissue dissociation may introduce artifacts, and cell specific fluorescent markers are not always available, limiting the use of flow sorting for cell procurement from native tissues [40]. In some instances, micropunching and microaspiration techniques have been applied for optimizing downstream applications [11], [12].

Here, we applied the current version of CTAS v.4.1 for microdissection of mouse and rat brains and demonstrated its ability to rapidly isolate desired tissue regions and even individual cells from both fresh frozen and live tissues. The procedure is simple, rapid and requires minimal training. Tissues are minimally treated and samples may be acquired from either fresh frozen or live brain samples (*CTAS-live*). Various ID of the DCU tip ensures precision that ranges from 20 µm (individual cells) to 100 µm (subanatomical regions). Adjustable vacuum strength ensure collection from tissue sections with various thickness (up to 500 µm) providing flexibility and capabilities to collect large sample volumes prerequisite in some downstream applications (e.g. 2D gels in proteomics studies). CTAS technology may be easily automated offering even wider range of cell- and tissue-specific separation parameters. Using CTAS, we successfully dissected individual neurons from mouse adult brain tissues demonstrating that the principle can provide single cell resolution similar to current laser-assisted technologies (Fig. 5). Acquisition of individual cells clearly requires careful optimization of microdissection parameters including proper selection of DCU ID, vacuum strength and duration. Higher vacuum strength may result in the collection of neighboring cells increasing contamination and extra care should be taken when working at the single cell level.

The quality of macromolecules isolated from CTAS dissected samples demonstrate their high integrity and applicability for further analyses (Figs. 7 and 8). This is perhaps one of the most important characteristics for applicability of microdissection technologies in experimental research. Multiple reports [23], [41], [42] and our past experience with laser-assisted technologies [15], [43] indicate that obtaining high quality RNA may not be a trivial task. Tissue fixation procedure and exposure to laser during the microdissection may both contribute to the increased rates of RNA degradation [23], [24]. Unlike laser-assisted instruments, CTAS operates with minimally treated tissues (fresh frozen, sucrose treated or live) and our data show that samples preserve high RNA quality even after two hours at room temperature (Fig. 7). RNA yield remained as expected and depended on the amount of collected tissue (Table 1).

Proteomic-based approaches aimed at the identification and investigation of protein markers in the actual histologically defined cell populations from heterogeneous tissues may lead to novel diagnostic, prognostic, or therapeutic markers that can be applied to monitor therapeutic toxicity or develop new therapies. Previously LCM was used in combination with 2D protein gels and peptide identification [44]. Our results show that the use of CTAS dissection allows successful collection of high quality tissue material suitable for proteomics using 2D electrophoresis and mass spectrometry analyses (Fig. 8). According to our observations, CTAS dissection is not associated with increased protein degradation. It may be due to the ability of CTAS to utilize live and freshly frozen tissues for dissection without prolonged exposures to elevated or room temperatures typical for many other dissection methods including LCM, thus minimizing protein degradation risk. However, due to a fairly large amount of sample material required for 2D gel electrophoresis, LCM-based sample acquisition utilizing on average 10 µm thin tissue sections might be a laborious and time consuming chore. Ability to dissect thicker tissue sections not affected by any fixation method is one of the advantages of vacuum-assisted CTAS microdissection.

Several brain-associated proteins including low abundant Neurocalcin-delta [45] have been successfully identified during 2D gels analysis of two neighboring areas of the mouse brain - DG and CA2–CA3 using DIA module of Decyder software (see Methods). Sequenced peptides represented the whole length of the corresponding proteins pointing to the lack of protein degradation (Dataset S1). DIA of the gels revealed differential expression of several brain-specific proteins, such as calretinin, neurocalcin-delta, and profillin-1 (Fig. 8). Although DIA is limited in regards to quantitative ability as compared to Biological Variability Analysis afforded by Difference Gel Electrophoresis (DIGE), the results of the current experiment indicate that CTAS-dissected tissues are perfectly suitable for further detailed DIGE analysis of multiple biological variants.

Another important advantage of CTAS over existing microdissection instruments is its ability to dissect native live tissues with minimal effect on cellular viability, which allows their further use as primary cultures. Here we demonstrated the use of CTAS for the procurement of neurogenic zones from both embryonic and adult rodent brains (Fig. 9). Rapid CTAS-based microdissection of brain neurogenic regions will significantly simplify current protocols aimed at cultivation of neural progenitors. It should be noted that culturing adult-derived neural progenitors (NPs) is not a trivial process and it may pose certain technical challenges due to the limited number of NPs and discrete regions of neurogenesis in the adult brain [46], [47], [48], [49]. Commonly used methods employ dissociation of whole brain tissues (e.g. hippocampus) and a density gradient to enrich for neural progenitors [29]. This protocol is time-consuming and cumbersome. Direct acquisition of neurogenic regions such as the subventriculrar zones (SVZs) can simplify the procedure and yield higher number of neurosphere colonies (Fig. 9).

Current report describes the application of CTAS for microdissection of mammalian brain tissues. Further work is needed to elucidate the precise parameters for microdissection of other tissue types. Clearly, depending on the tissue type, vacuum strength and duration may vary greatly and depending on the tissue’s extracellular matrix microdissection may be challenging and even demand for a specific tissue pretreatment. Current setup does not permit FFPE tissue dissection and further optimization of CTAS is necessary to overcome this methodological challenge. Other challenges include the choice of DCU, which may become clogged if acquired areas greatly exceed its inner diameter. At this point, the procedure for DCU production has been developed and each unit is carefully examined for consistency and uniformity of the tip surface. Tissue adherence to the surface of the capillary does not affect the overall dissection process as long as the dissected tissue section is kept moist, which may be ensured by occasional addition of a buffer (e.g. PBS or Hank’s solution). Resolution of CTAS is mainly controlled by the DCU ID ranging from several to hundreds of micrometers.

In conclusion, we have developed a versatile, efficient and cost-effective benchtop technology that ensures isolation of the specific tissue regions and cells from native and fresh frozen brain tissues. Both all purpose CTAS and CTAS-live specifically tailored for handling live cells will be available as prefabricated systems with dedicated and modified inverted microscopes. The systems are amenable to further automation. Although current CTAS is operated in transmitted light, it is also compatible with epifluorescence mode, permitting the collection of specifically-labeled cell populations or brain areas. Collected tissues and cells can be used in various downstream applications covering a wide range of techniques used in modern molecular biology. DNA, RNA, and protein qualities are not compromised in the samples collected with CTAS, making this method ideal for region and cell specific analyses using genomic and proteomic approaches.

## Supporting Information

Figure S1 **Collection of subventricular zones (SVZ) from the adult mouse brain.** Native coronal brain tissue section before (**A**) and after (**B**) dissection. Tissue thickness  = 300 µm. Scale bar  = 250 µm.
(EPS)

Dataset S1 **Representative MASCOT Peptide Mass Fingerprinting (PMF) search results for PP2AB, SPEE, HS90B and CALB2 demonstrate that identified peptides are evenly distributed over the whole length of the corresponding proteins pointing to the lack of protein degradation during CTAS-based microdissection procedure.**
(PDF)
