# Supplementary material for: Novel Cell and Tissue Acquisition System (CTAS): Microdissection of Live and Frozen Brain Tissues
Source: PLoS One. 2012 Jul 24;7(7):e41564. doi: 10.1371/journal.pone.0041564 (PMC3404047; doi:10.1371/journal.pone.0041564)
Supplement: Dataset S1 — Representative MASCOT Peptide Mass Fingerprinting (PMF) search results for PP2AB, SPEE, HS90B and CALB2 demonstrate that identified peptides are evenly distributed over the whole length of the corresponding proteins pointing to the lack of protein degradation during CTAS-based microdissection procedure. (PDF) [file pone.0041564.s002.pdf]

Mascot Search Results

Protein View

Match to: PP2AB_MOUSE Score: 63 Expect: 0.012
Serine/threonine-protein phosphatase 2A catalytic subunit beta isoform OS=Mus musculus GN=Ppp2cb PE=1 SV=1

Nominal mass (M_r): 36123; Calculated pI value: 5.21
NCBI BLAST search of PP2AB_MOUSE against nr
Unformatted sequence string for pasting into other applications

Taxonomy: Mus musculus

Fixed modifications: Carbamidomethyl (C)
Variable modifications: Oxidation (M)
Cleavage by Trypsin: cuts C-term side of KR unless next residue is P
Number of mass values searched: 55
Number of mass values matched: 8
Sequence Coverage: 36%

Matched peptides shown in **Bold Red**

```
  1 MDDKAFTKEL DQWVEQLNEC KQLNENQVRT LCEKAKEILT KESNVQEVRC
 51 PVTVCGDVHG QFHDLMELFR IGGKSPDTNY LFMGDYVDRG YYSVETVTLL
101 VALKVRYPER ITILRGNHES RQITQVYGFY DECLRKYGNA NVWKYFTDLF
151 DYLPLTALVD GQIFCLHGGL SPSIDTLDHI RALDRLQEVP HEGPMCDLLW
201 SDPDDRGGWG ISPRGAGYTF GQDISETFNH ANGLTLVSRA HQLVMEGYNW
251 CHDRNVVTIF SAPNYCYRCG NQAAIMELDD TLKYSFLQFD PAPRRGEPHV
301 TRRTPDYFL
```

Show predicted peptides also

Sort Peptides By ◉ Residue Number ○ Increasing Mass ○ Decreasing Mass

Start - End	Observed	Mr(expt)	Mr(calc)	Delta	Miss	Sequence
22 - 29	1000.5300	999.5227	999.5097	0.0130	0	K.QLNENQVR.T
75 - 89	1808.7482	1807.7409	1807.7723	-0.0314	0	K.SPDTNYLFMGDYVDR.G Oxidation (M)
90 - 106	1910.9869	1909.9797	1910.0877	-0.1080	1	R.GYYSVETVTLLVALKVR.Y
122 - 135	1791.8034	1790.7962	1790.8298	-0.0336	0	R.QITQVYGFYDECLR.K
137 - 144	951.4625	950.4553	950.4610	-0.0057	0	K.YGNANVWK.Y
215 - 239	2655.2424	2654.2351	2654.2725	-0.0374	0	R.GAGYTFGQDISETFNHANGLTLVSR.A
255 - 268	1703.7961	1702.7888	1702.8137	-0.0249	0	R.NVVTIFSAPNYCYR.C
284 - 294	1340.6349	1339.6276	1339.6561	-0.0285	0	K.YSFLQFDPAPR.R

Mascot Search Results

Protein View

Match to: SPEE_MOUSE Score: 69 Expect: 0.003
Spermidine synthase OS=Mus musculus GN=Srm PE=2 SV=1

Nominal mass (M_r): 34543; Calculated pI value: 5.31
NCBI BLAST search of SPEE_MOUSE against nr
Unformatted sequence string for pasting into other applications

Taxonomy: Mus musculus

Fixed modifications: Carbamidomethyl (C)
Variable modifications: Oxidation (M)
Cleavage by Trypsin: cuts C-term side of KR unless next residue is P
Number of mass values searched: 33
Number of mass values matched: 7
Sequence Coverage: 26%

Matched peptides shown in **Bold Red**

```
  1 MEPGPDGPAA PGPAAIREGW FRETCSLWPG QALSLQVEQL LHHRRSRYQD
 51 ILVFRSKTYG NVLVLDGVIQ CTERDEFSYQ EMIANLPLCS HPNPRKVLII
101 GGGDGGVLRE VVKHPSVESV VQCEIDEDVI EVSKKFLPGM AVGFSSSKLT
151 LHVGDGFEFM KQNQDAFDVI ITDSSDPMGP AESLFKESYY QLMKTALKED
201 GILCCQGECQ WLHLDLIKEM RHFCKSLFPV VDYAYCSIPT YPSGQIGFML
251 CSKNPSTNFR EPVQQLTQAQ VEQMQLKYYN SDMHRAAFVL PEFTRKALND
301 IS
```

Show predicted peptides also

Sort Peptides By ◉ Residue Number ○ Increasing Mass ○ Decreasing Mass

Start - End	Observed	Mr(expt)	Mr(calc)	Delta	Miss	Sequence
1 - 22	2279.0984	2278.0911	2278.0953	-0.0042	1	-.MEPGPDGPAAPGPAAIREGWFR.E
48 - 55	1053.5476	1052.5403	1052.5655	-0.0252	0	R.YQDILVFR.S
58 - 74	1936.9450	1935.9378	1935.9724	-0.0347	0	K.TYGNVLVLDGVIQCTER.D
96 - 109	1353.7764	1352.7691	1352.8140	-0.0449	1	R.KVLIIGGGDGGVLR.E
97 - 109	1225.7058	1224.6985	1224.7190	-0.0206	0	K.VLIIGGGDGGVLR.E
278 - 295	2233.0896	2232.0823	2232.0422	0.0401	1	K.YYNSDMHRAAFVLPEFTR.K Oxidation (M)
286 - 295	1150.6021	1149.5948	1149.6182	-0.0234	0	R.AAFVLPEFTR.K

{MATRIX SCIENCE} Mascot Search Results

Protein View

Match to: HS90B_MOUSE Score: 71 Expect: 0.0021
Heat shock protein HSP 90-beta OS=Mus musculus GN=Hsp90ab1 PE=1 SV=2

Nominal mass (M_r): 83615; Calculated pI value: 4.97
NCBI BLAST search of HS90B_MOUSE against nr
Unformatted sequence string for pasting into other applications

Taxonomy: Mus musculus

Fixed modifications: Carbamidomethyl (C)
Variable modifications: Oxidation (M)
Cleavage by Trypsin: cuts C-term side of KR unless next residue is P
Number of mass values searched: 71
Number of mass values matched: 12
Sequence Coverage: 23%

Matched peptides shown in **Bold Red**

```
  1 MPEEVHHGEE EVETFAFQAE IAQLMSLIIN TFYSNKEIFL RELISNASDA
 51 LDKIRYESLT DPSKLDSGKE LKIDILPNPQ ERTLTLVDTG IGMTKADLIN
101 NLGTIAKSGT KAFMEALQAG ADISMIGQFG VGFYSAYLVA EKVVVITKHN
151 DDEQYAWESS AGGSFTVRAD HGEPIGRGTK VILHLKEDQT EYLEERRVKE
201 VVKKHSQFIG YPITLYLEKE REKEISDDEA EEEKGEKEEE DKEDEEKPKI
251 EDVGSDEEDD SGKDKKKKTK KIKEKYIDQE ELNKTKPIWT RNPDDITQEE
301 YGEFYKSLTN DWEDHLAVKH FSVEGQLEFR AFLFIPRRAP FDLFENKKKK
351 NNIKLYVRRV FIMDSCDELI PEYLNFIRGV VDSEDLPLNI SREMLQQSKI
401 LKVIRKNIVK KCLELFSELA EDKENYKKFY EAFSKNLKLG IHEDSTNRRR
451 LSELLRYHTS QSGDEMTSLS EYVSRMKETQ KSIYYITGES KEQVANPAFV
501 ERVRKRGFEV VYMTEPIDEY CVQQLKEFDG KSLVSVTKEG LELPEDEEEK
551 KKMEESKAKF ENLCKLMKEI LDKKVEKVTI SNRLVSSPCC IVTSTYGWTA
601 NMERIMKAQA LRDNSTMGYM MAKKHLEINP DHPIVETLRQ KAEADKNDKA
651 VKDLVVLLFE TALLSSGFSL EDPQTHSNRI YRMIKLGLGI DEDEVTAEEP
701 SAAVPDEIPP LEGDEDASRM EEVD
```

Show predicted peptides also

Sort Peptides By ◉ Residue Number ○ Increasing Mass ○ Decreasing Mass

Start - End	Observed	Mr(expt)	Mr(calc)	Delta	Miss	Sequence
73 - 82	1194.6427	1193.6354	1193.6404	-0.0050	0	K.IDILPNPQER.T
149 - 168	2256.0970	2255.0898	2254.9516	0.1382	0	K.HNDDEQYAWESSAGGSFTVR.A
205 - 219	1808.9492	1807.9419	1807.9509	-0.0090	0	K.HSQFIGYPITLYLEK.E
320 - 330	1348.6543	1347.6471	1347.6572	-0.0101	0	K.HFSVEGQLEFR.A
320 - 337	2193.0609	2192.0536	2192.1531	-0.0995	1	K.HFSVEGQLEFRAFLFIPR.R
338 - 347	1236.6249	1235.6177	1235.6299	-0.0122	1	R.RAPFDLFENK.K
360 - 378	2390.1382	2389.1309	2389.1334	-0.0025	0	R.VFIMDSCDELIPEYLNFIR.G Oxidation (M)
379 - 392	1513.7753	1512.7680	1512.7784	-0.0103	0	R.GVVDSEDLPLNISR.E
613 - 623	1264.6385	1263.6312	1263.4934	0.1379	0	R.DNSTMGYMMAK.K Oxidation (M)
624 - 639	1911.0228	1910.0155	1910.0374	-0.0218	1	K.KHLEINPDHPIVETLR.Q
625 - 639	1782.9488	1781.9416	1781.9424	-0.0008	0	K.HLEINPDHPIVETLR.Q
686 - 719	3535.6461	3534.6388	3534.6373	0.0015	0	K.LGLGIDEDEVTAEEPSAAVPDEIPPLEGDEDASR.M

Mascot Search Results

Protein View

Match to: CALB2_MOUSE Score: 55 Expect: 0.081
Calretinin OS=Mus musculus GN=Calb2 PE=1 SV=3

Nominal mass (M_r): 31467; Calculated pI value: 4.94
NCBI BLAST search of CALB2_MOUSE against nr
Unformatted sequence string for pasting into other applications

Taxonomy: Mus musculus

Fixed modifications: Carbamidomethyl (C)
Variable modifications: Oxidation (M)
Cleavage by Trypsin: cuts C-term side of KR unless next residue is P
Number of mass values searched: 41
Number of mass values matched: 7
Sequence Coverage: 32%

Matched peptides shown in **Bold Red**

```
  1 MAGPQQQPPY LHLAELTASQ FLEIWKHFDA DGNGYIEGKE LENFFQELEK
 51 ARKGSGMMSK SDNFGEKMKE FMQKYDKNSD GKIEMAELAQ ILPTEENFLL
101 CFRQHVGSSA EFMEAWRKYD TDRSGYIEAN ELKGFLSDLL KKANRPYDEP
151 KLQEYTQTIL RMFDLNGDGK LGLSEMSRLL PVQENFLLKF QGMKLTSEEF
201 NAIFTFYDKD GSGYIDENEL DALLKDLYEK NKKEMNIQQL TTYRKSVMSL
251 AEAGKLYRKD LEIVLCSEPP V
```

Show predicted peptides also

Sort Peptides By ◉ Residue Number ○ Increasing Mass ○ Decreasing Mass

Start - End	Observed	Mr(expt)	Mr(calc)	Delta	Miss	Sequence	
40 - 50	1425.6827	1424.6755	1424.6823	-0.0069	0	K.ELENFFQELEK.A	
83 - 103	2553.2423	2552.2350	2552.2654	-0.0304	0	K.IEMAELAQILPTEENFLLCFR.Q	Oxidation (M)
104 - 117	1650.8507	1649.8434	1649.7256	0.1178	0	R.QHVGSSAEFMEAWR.K	Oxidation (M)
134 - 142	1020.5924	1019.5851	1019.6015	-0.0164	1	K.GFLSDLLKK.A	
152 - 161	1264.6918	1263.6845	1263.6823	0.0022	0	K.LQEYTQTILR.M	
179 - 189	1313.7619	1312.7546	1312.7755	-0.0208	0	R.LLPVQENFLLK.F	
234 - 244	1412.6853	1411.6781	1411.6765	0.0015	0	K.EMNIQQLTTYR.K	Oxidation (M)
